# Pediatric Stroke in Asia and Africa: Epidemiology, Etiology, and Resource-Stratified Care Across Geographic and System-Level Disparities

**DOI:** 10.1177/11795735261478000

**Published:** 2026-08-12

**Authors:** Anne Francine Pino, Afton Widdershins, Gayatra Mainali

**Affiliations:** 1 Pennsylvania State College of Medicine, Hershey, PA, USA; 2 Division of Child Neurology, Department of Pediatrics, Penn State Health Children’s Hospital, 12310 Penn State College of Medicine, Hershey, PA, USA

**Keywords:** global health, pediatric stroke, stroke

## Abstract

Pediatric stroke is a rare but serious cause of morbidity and mortality, with disproportionately high burden in low- and middle-income countries (LMICs). Asia and Africa report the highest global incidence and prevalence, driven by region-specific risk factors such as sickle cell disease (SCD), moyamoya disease (MMD), and infectious etiologies. Despite this substantial burden, data from these regions remain limited and fragmented, hindering effective clinical care and policy development. Incidence rates across Asia and Africa consistently exceed those in high-income countries. Western Sub-Saharan Africa, Southeast Asia, and Central Asia present some of the world’s highest age-standardized rates. The most common etiologies include infections, particularly tuberculous meningitis, hematologic disorders such as SCD, and vasculopathies including MMD. Clinical features resemble those in high-income settings; however, children in LMICs experience delays in diagnosis due to limited diagnostic modalities, shortages of pediatric stroke expertise, and lack of standardized protocols. Management practices vary widely, consistent with global challenges due to lack of clinical data. LMICs are further constrained by resource limitations. Pediatric stroke care disparities in Asia and Africa reflect both geographic inequities between LMICs and HICs and system-level inequities within LMICs, where tertiary urban centers may provide advanced care while rural and secondary facilities remain limited. Emerging initiatives, including resource-adapted diagnostic algorithms, community screening tools, educational programs, and multicenter prevention trials, highlight growing efforts to strengthen pediatric stroke care. Improving outcomes require strengthening epidemiological surveillance, expanding regional and national stroke registries, developing resource-adapted diagnostic and treatment guidelines, and increasing access to preventive and therapeutic interventions. This review synthesizes available literature on pediatric stroke across Asian and African regions using the Global Burden of Disease (GBD) regional framework. Countries within Sub-Saharan Africa, North Africa and the Middle East, East Asia, High-Income Asia Pacific, South Asia, and Southeast Asia were examined. Key themes include epidemiology, etiologies, clinical presentations, and management strategies within these regions.

## Graphical abstract

**Graphical Abstract:**
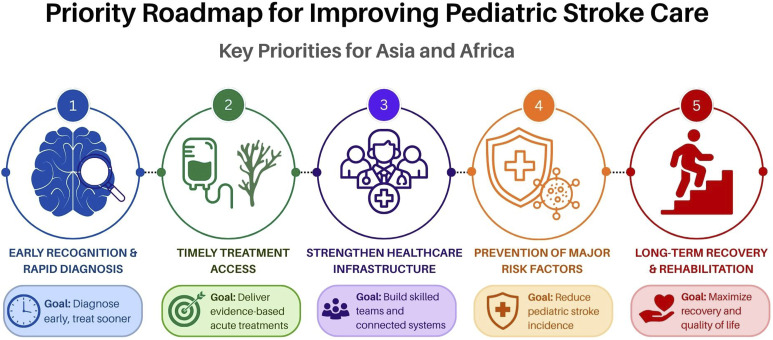

## 1. Introduction

Pediatric stroke is a rare but critical, and often under-recognized, contributor to morbidity and mortality among children worldwide. It is characterized by acute onset of focal neurological deficits and commonly presents as seizures or non-focal findings such as altered mental status, headaches, and episodes of transient weakness. Symptoms of stroke in children may also be subtle; therefore, a high index of suspicion is required, as it can be easily missed, contributing to diagnostic delay and challenges. Timely diagnosis remains a challenge in many settings due to non-specific presentations and variability in clinical suspicion across providers and health systems. In children, stroke typically occurs in the setting of underlying conditions, rather than atherosclerotic mechanisms as seen in adults. Emerging evidence suggests that thrombus composition and pathophysiology in children differ from adults, complicating the direct translation of adult thrombolysis data to pediatric practice and contributing to ongoing controversy surrounding acute reperfusion therapies in children. Lack of trials and evidence-based pediatric data contributes to variability in management.

Marked global disparities exist in the burden of pediatric stroke, with low- and middle-income countries (LMICs) experiencing disproportionately higher incidence, prevalence, and mortality compared to high-income countries (HICs). The highest documented incidences of pediatric stroke worldwide occur in Asian and African regions, driven in part by the high prevalence of conditions such as Sickle Cell Disease (SCD), Moyamoya Disease (MMD), and central nervous system infections. Importantly, Asia and Africa are not monolithic regions; both include tertiary centers with advanced imaging and subspecialty care alongside rural and secondary facilities with limited diagnostic and treatment capacity. As a result, much of the published literature reflects data from tertiary-care centers, representing high-resource settings within otherwise resource-limited regions, rather than population-level patterns. Therefore, resource limitation should be understood as a spectrum within and across countries rather than a purely geographic designation.

Despite this substantial burden, available data from these regions remain limited and fragmented, hindering a comprehensive understanding of pediatric stroke patterns. These limitations are driven by the lack of national stroke registries, variable reporting practices, and underrepresentation of rural populations. Additionally, publication bias and the exclusion of non-indexed or locally published data further contribute to gaps in the literature. These disparities are further compounded by delayed diagnosis, limited access to advanced imaging, and shortages of pediatric stroke expertise across many settings.

According to the Global Burden of Disease regional framework, countries examined include: regions within Sub-Saharan Africa, North Africa and the Middle East, East Asia, High-Income Asia Pacific, South Asia, and Southeast Asia This review aims to synthesize the current evidence on pediatric stroke care in Asian and African regions by comparing regional epidemiology, delineating etiologic patterns, and evaluating variations in clinical management, with the objective of identifying geographic and system-level disparities and highlighting actionable priorities for improving care in resource-limited settings.

## 2. Methods

### 2.1. Study Design & Search Strategy

We performed a narrative review using a structured literature search approach. A narrative review approach was selected because available studies were highly heterogeneous in design, population, definitions, regional representation, and reported outcomes, limiting suitability for meta-analysis. A comprehensive search was conducted using PubMed/MEDLINE, Embase, and Scopus to identify relevant articles reporting on pediatric stroke care in Asian and African countries or regions, published between 1994 and 2025. A combination of keywords and Medical Subject Headings (MeSH) terms was used, including “pediatric stroke,” “Asia,” “Africa,” “low- and middle-income countries,” and “ischemic” or “hemorrhagic stroke.”

Studies were eligible for inclusion if they reported on pediatric stroke epidemiology, etiology, diagnosis, or management within Asian or African regions or in LMIC settings. Exclusion criteria included non-English publications, adult-only studies, and articles not relevant to pediatric stroke. Eligible study designs included observational studies (retrospective and prospective), cohort studies, systematic and scoping reviews, clinical guidelines, and meta-analyses.

Titles and abstracts were screened for relevance, followed by full-text review of selected articles. Data were synthesized descriptively to identify patterns in epidemiology, etiologies, clinical presentation, and management across regions. Additionally, a formal risk of bias assessment was not conducted, and findings should be interpreted in the context of these limitations.

## 3. Epidemiology & Regional Burden

### 3.1. Global Burden

The global burden of pediatric stroke in 2021 was estimated at 11.8 per 100,000 children and adolescents, with LMICs accounting for 81.6% of incident cases.^1^ Incidence rates remain markedly higher in LMICs compared with HICs with a rate of 13.5 per 100,000 vs 10.0 per 100,000, respectively.^1^ Regions with the highest pediatric stroke incidence include Western Sub-Saharan Africa, North Africa and the Middle East, Oceania, the Caribbean, and Southeast Asia, although robust epidemiologic data remain limited across many of these settings. For comparison, the pediatric stroke incidence in the United States is substantially lower, estimated at 2.3–2.7 per 100,000 children per year.^2^ The development of heightened suspicion for pediatric stroke in HICs, especially those with access to high-quality MRIs and abundant healthcare resources, aims to decrease the overall stroke burden, measured by mortality and death rates of ischemic stroke among children. Despite anticipated decreases in global mortality, overall incidence and prevalence are projected to rise, disproportionately affecting LMICs.^1^ Generating accurate regional estimates, particularly across Africa and Asia, remains challenging due to the vast geographic and socioeconomic diversity within these regions.

Stroke rates also vary by age and stroke subtype. Globally, ischemic strokes are predominant. A 2013 GBD analysis reported that LMICs bear a significantly higher burden as the prevalence of ischemic stroke was 4-to-5 fold higher and hemorrhagic stroke about 2-fold higher in developing countries.^1,3^ By age, a study focusing on resource limited settings reported rates of 10.2 per 100,000 in neonates (birth to 28 days) and 1.72 per 100,000 among children (29 days to 19 years).^3^ Regional variation in pediatric stroke burden is summarized in Table 1, highlighting substantial heterogeneity across subregions.

**Table 1.** Epidemiology and Regional Burden of Pediatric Stroke in Asia and Africa

Region/Subregion	Incidence/Prevalence	Key findings	Representative sources
Global	∼11.8 per 100,000 children (2021)	LMICs account for ∼81.6% of incident cases; higher burden vs HICs	GBD 2021
LMIC vs HIC comparison	LMIC: ∼13.5 per 100,000 vs HIC: ∼10.0 per 100,000	Higher incidence, prevalence, and mortality in LMICs	GBD studies
Western Sub-Saharan Africa	Among highest global ASRs	Second highest global incidence; major burden driven by SCD	GBD 2024
Sub-Saharan Africa (overall)	Variable; hospital-based data up to 16 per 100,000	High burden; limited population-level data; strong SCD contribution	Kenya, Cameroon studies
North Africa & Middle East	∼1.8–13 per 100,000 (country-specific)	Variable incidence; influenced by cardiac disease, infections, and genetic factors	Iran, Saudi Arabia, Jordan
Eastern & Southern Africa	High prevalence of SCD-related stroke	Up to 2.9–16.9% stroke prevalence in children with SCD	Multi-country SCD studies
South Asia	Elevated incidence; limited national data	High burden linked to infections, anemia, and prothrombotic states	India, Bangladesh
Southeast Asia	Among highest global incidence regions	High burden in countries like Myanmar and Laos; infection-driven stroke common	GBD, regional studies
East Asia	∼2.3–6.4 per 100,000 (country-specific)	Lower than LMIC averages but still elevated vs HICs; MMD prominent	China, Taiwan
High-Income Asia Pacific	Lower relative incidence	Better detection and reporting; advanced stroke systems	Regional registry data

Abbreviations: LMIC = low- and middle-income countries; HIC = high-income countries; SCD = sickle cell disease; MMD = moyamoya disease; ASR = age-standardized rate; GBD = Global Burden of Disease.

### 3.2. Africa

Broad-scale evaluations of pediatric stroke (ages < 20 years) from the 2024 Global Burden Disease study show that Africa has some of the highest pediatric stroke rates globally, second only to Asia. Western Sub-Saharan Africa demonstrated the highest age-standardized rates (ASRs) and ranked second worldwide in total pediatric stroke incidence in 2021. Other Sub-Saharan regions, along with North Africa and the Middle East have similarly high ASRs.^1^

Institution-based data similarly underscores a substantial burden. A retrospective cross-sectional study of a teaching center in Kenya identified 32 cases of pediatric stroke over 5 years corresponding to an estimated prevalence of 16 per 100,000, higher than LMICs estimates, indicating that pediatric stroke is not uncommon in Kenyan populations.^4^ In Cameroon, an analysis of children ≤15 years old documented an incidence of 1.82 per 1,000 pediatric hospitalizations.^5^ Data from Middle Eastern countries show similar variability: the incidence of pediatric ischemic stroke is 1.8 per 100,000 in Iran’s Khorasan Province, while Saudi Arabia reports an annual hospital-based frequency of 27.1 per 100,000 among children aged 1 month to 12 years.^6,7^ Jordan likewise reports incidence rates of 8-13 per 100,000 among children aged 5-14 years.^8^

Sickle cell disease (SCD) is a major contributor to the pediatric stroke burden in Africa. The first systematic review that analyzed neurologic events in African children with SCD reported a stroke prevalence of 4.2% in a pooled sample of 18,977 children obtained from 23 studies.^9^ Sub-Saharan Africa (SSA) accounts for approximately 80% of global SCD cases, with stroke prevalence in affected children ranging from 2.9% to 16.9%. The results from this study: Nigeria (2.9% in 2013), Cameroon (6.7% in 2013), Malawi (8.5% in 2015), Tanzania (16.9% in 2012), and Uganda (6.2% in 2012-2014).^10-12^ A prospective cohort of 224 Tanzanian children with sickle cell anemia found silent cerebral infarcts in 27% of neurologically asymptomatic participants, underscoring the high burden of subclinical disease.^13^ In contrast, SCD-related stroke prevalence in Kuwait (1.4%) and Saudi Arabia (2%) is lower, reflecting differences in genetic variants, screening programs, and healthcare infrastructure.^11^

Collectively, these findings demonstrate that pediatric stroke incidence in African regions consistently exceeds rates observed in high-income countries, with SCD serving as a major and regionally distinctive driver of stroke risk.

### 3.3. Asia

The epidemiology of pediatric stroke across Asia is difficult to characterize due to wide geographic variability and limited country-level data. A 2021 comprehensive analysis reported that Southeast Asia and Central Asia have among the highest pediatric stroke incidences worldwide, with unexpectedly elevated burdens in countries such as Myanmar and the Lao People’s Democratic Republic.^1^ Several nations, including Indonesia, China, and Taiwan, have undertaken multicenter or national investigations to better define their local epidemiology. In Indonesia, reported pediatric stroke occurrence ranges from 5.9% to 9.08%.^14^ In China, point prevalence and annual incidence estimates are 4.82 per 100,000 and 2.34 per 100,000, respectively.^15^ Taiwan’s 2018 analysis documented a two-year prevalence of 14.2 per 100,000 and a 2011 incidence of 6.4 per 100,000.^2^

Across most Asian regions, ischemic stroke predominates, though exceptions exist. A regional study in Indonesia found hemorrhagic stroke to be more common, particularly among males and older children, likely reflecting higher rates of trauma and unintentional injuries.^14^

Distinct region-specific disease patterns also contribute to the variability in pediatric stroke across Asia. Moyamoya disease, for example, remains a major etiology in East Asian populations and is particularly prominent in Japan, where prevalence is estimated at 3.16-10.5 per 100,000 and incidence at 0.35-0.94 per 100,000, with up to 80% of pediatric moyamoya cases presenting with ischemic events.^16^ These elevated rates underscore the influence of genetic, environmental, and population-specific risk factors, and highlight subregions that would benefit from targeted research and surveillance.

Although incidence estimates vary widely across the Asian subregions, reported rates generally remain higher than those observed in developed countries. These epidemiologic trends demonstrate the need for standardized methodologies, larger-scale multicenter studies, and robust national surveillance systems to more accurately delineate the pediatric stroke burden across Asia.

## 4. Etiologies & Risk Factors

### 4.1. Pediatric Stroke Classification

**Figure 1.**
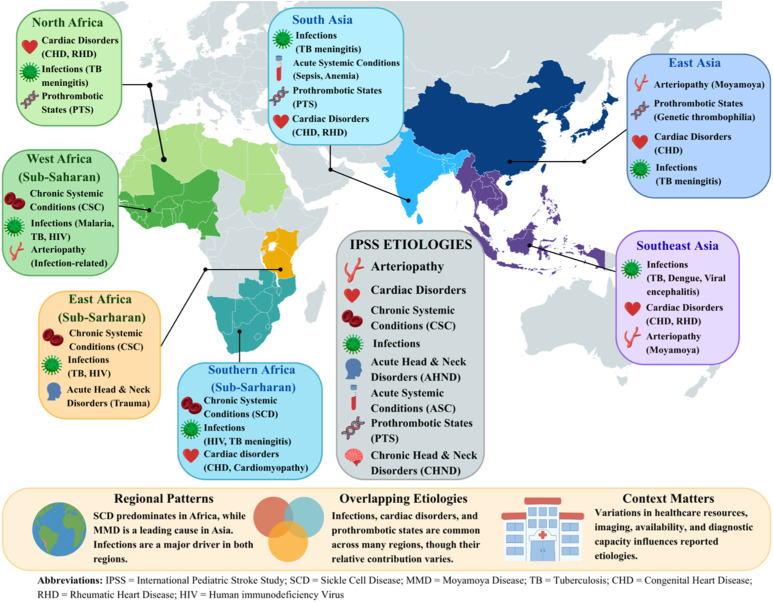
Dominant etiologies of pediatric stroke in Asia and Africa (*IPSS Classification*)

Etiologies and risk factors for pediatric stroke can be classified according to the International Pediatric Stroke Study (IPSS) framework, which includes arteriopathy, cardiac disorders, chronic systemic conditions (CSCs), infections, acute head and neck disorders (AHNDs), acute systemic conditions (ASCs), prothrombotic states (PTSs), and chronic head and neck disorders (CHNDs).^17^ This framework is particularly useful in Asia and Africa because dominant etiologies often reflect preventable or regionally concentrated conditions, including infection, SCD, and MMD. Identifying these underlying factors is essential, as 77-79% of childhood stroke cases have at least one preexisting risk factor, with patterns varying by age and ethnicity.^18^ A clear understanding of region-specific etiologies not only enhances diagnostic suspicion but also informs prevention strategies and targeted management of underlying conditions in these high-burden settings. Etiologies and risk factors, categorized according to the IPSS framework, are summarized in Table 2, highlighting region-specific patterns across Asia and Africa (Figure 1).

**Table 2.** IPSS-Based Classification of Pediatric Stroke Etiologies in Asia and Africa

IPSS category	Description	Common examples in Africa	Common examples in Asia	Key notes/Patterns
Arteriopathy	Structural or inflammatory abnormalities of cerebral vessels	Infection-related vasculopathy (TB meningitis, HIV-associated vasculopathy), moyamoya secondary to SCD	Moyamoya disease (high prevalence in East Asia), arterial dissection, mineralizing angiopathy	Likely underdiagnosed in LMICs due to limited vascular imaging
Cardiac Disorders	Congenital or acquired heart disease leading to embolic stroke	Rheumatic heart disease, congenital heart disease, cardiomyopathy	Congenital heart disease, endocarditis, cardiomyopathy	RHD remains a major contributor in LMICs
Chronic Systemic Conditions (CSC)	Chronic diseases increasing stroke risk	Sickle cell disease (major contributor), chronic anemia	Thalassemia, iron deficiency anemia	SCD accounts for substantial stroke burden in Sub-Saharan Africa
Infections	CNS or systemic infections leading to stroke	Tuberculous meningitis, HIV, bacterial meningitis, malaria	Tuberculous meningitis, viral encephalitis, bacterial meningitis	Most common etiology in many LMIC settings; largely preventable
Acute Head & Neck Disorders (AHND)	Trauma or acute vascular injury	Head trauma, arterial dissection	Trauma-related dissection, infection-related vasculopathy	Often underreported; variable by region
Acute Systemic Conditions (ASC)	Acute illness causing systemic instability	Sepsis, dehydration, severe anemia	Sepsis, dehydration, inflammatory states	Frequently coexists with infection-related stroke
Prothrombotic States (PTS)	Inherited or acquired thrombophilia	Protein C/S deficiency, antithrombin deficiency, HIV-related hypercoagulability	Factor V Leiden, MTHFR polymorphisms, protein C/S deficiency	Prevalence varies widely; may be underdiagnosed in LMICs
Chronic Head & Neck Disorders (CHND)	Chronic structural abnormalities	SCD-related vasculopathy, prior infections	Moyamoya syndrome, prior radiation exposure	Overlaps with arteriopathy; region-specific patterns

Abbreviations: IPSS = International Pediatric Stroke Study; SCD = sickle cell disease; TB = tuberculosis; CNS = central nervous system; CSC = chronic systemic conditions; AHND = acute head and neck disorders; ASC = acute systemic conditions; PTS = prothrombotic states; CHND = chronic head and neck disorders.

### 4.2. Common Causes

#### 4.2.1. Infectious

In developing countries, infection is among the most common causes of childhood strokes. Several Asian studies report intracranial infections in up to 56% of pediatric stroke cases.^19^ According to Prevalence of Acute critical Neurological disease in children: a Global Epidemiological Assessment (PANGEA), central nervous system infection was the most common neurological insult among critically ill children in Africa (50%) and Asia (36%).^11^ Similar trends have been documented across tertiary centers in Saudi Arabia, Northern India, Beijing, and Turkey, where infections consistently rank as the most frequent risk factor for pediatric stroke.^18^ In Bangladesh, infection was present in 40.5% of children with stroke.^11^ A range of infectious diseases contribute to this burden, including meningitis, HIV, and rheumatic heart disease, all of which remain prevalent in many Asian and African regions, contributing to their stroke burden and overall morbidity and mortality.^20^ Among infectious etiologies, tuberculous meningitis (TBM) is the most common cause of pediatric stroke, as demonstrated by studies from India, Turkey, and China.^21-24^ In Vietnam and South Africa, infarcts occurred in 30% and 32% of children with TBM, and in Turkey, TBM accounted for 26% of CNS infections associated with acute ischemic stroke. TBM accounted for 78% and 50% of infection-associated arteriopathies in North and Eastern India.^11^ This high burden is expected given that Southeast Asia and SSA have the first- and second-highest tuberculosis prevalence globally, and TBM frequently results in vasculitis and subsequent infarction.^20,25^ A study in SSA found that 40% of children with bacterial meningitis had transcranial Doppler evidence of cerebral hypoperfusion, later developing motor deficits consistent with infarction.^11^ *Streptococcus pneumonia* and *Haemophilus influenza* are other pathogens that contribute to stroke burden and reflect gaps in vaccination coverage in many LMICs.

Human immunodeficiency virus (HIV) is another important risk factor for pediatric stroke in African regions. Nearly half of all new pediatric HIV infections occur in seven African countries, and two-thirds of children living with HIV reside in Eastern and Southern Africa.^26^ HIV infection was identified in 9.4% of childhood stroke cases in Kenya, and a Nairobi study found HIV and other infections to be the third and fourth most common risk factors.^4,11^ High rates of pediatric HIV infections increase vulnerability to opportunistic infections, susceptibility to serious complications, and risk of stroke secondary to HIV-driven inflammatory vasculopathy.^27-29^ Infectious diseases are a major and preventable contributor to pediatric stroke in Asia and Africa. Strengthening vaccination programs, improving early diagnosis and treatment of CNS infections, and expanding HIV care are essential components of effective stroke prevention strategies in LMICs.

#### 4.2.2. Hematologic

SCD is one of the greatest contributors to pediatric stroke in Africa, with estimated incidence between 2.9% and 5.8% of sub-Saharan African sickle cell patients suffering from stroke.^9,12,30,31^ As the most common inherited disorder in SSA and a leading cause of neurological morbidity and mortality, SCD accounts for a substantial proportion of childhood cerebrovascular disease. More than 60,000 children in SSA experience SCD-related stroke, with Nigerians accounting for nearly half of all cases.^12,20^ The pathophysiology of SCD itself predisposes children to ischemic and hemorrhagic events while also making them more vulnerable to strokes secondary to anemia and infectious pathologies like malaria and HIV.^12,32,33^

Regional studies highlight additional cerebrovascular complications. In Tanzania, 27% of 224 children with SCD had silent cerebral infarcts, reflecting a high burden of subclinical vasculopathy.^13^ Genetic modifiers and co-occurring hemoglobinopathies further influence stroke risk. Polymorphisms in heme oxygenase-1 and components of the renin–angiotensin system have been associated with increased risk in Egyptian children.^34,35^ Another Egyptian study did not find an association between higher stroke risk and a specific SCD ß-globin haplotype.^36^ Co-inherited α-thalassemia may confer protection, as observed in Angolan children.^37^ A study from Sudan also identified high frequencies of SCD across Afro-Asiatic, Nilo-Saharan, and Niger-Congo tribes, underscoring the wide geographic and ethnic distribution of the disease.^38^ Overall, SCD represents one of the most devastating and prevalent causes of pediatric stroke globally, with its impact felt most acutely in African countries.

In addition to SCD, iron deficiency anemia (IDA) is an important and often underrecognized hematologic risk factor for pediatric arterial ischemic stroke (AIS) in Asia and Africa. Anemia affects 58.8% of children aged 6-59 months in low-income countries (LICs) and 50% in LMICs. IDA was a common AIS risk factor in China (16.7%) and India (10.5%), and an Egyptian study found IDA in 57.1% of pediatric stroke cases, compared with 26% of controls.^11^ It has also been associated with ischemic stroke in India and Thailand. In Thailand, 39.2% of AIS cases had IDA.^39^ Additional relevant hemoglobinopathies include thalassemia, frequently reported in China and other Asian regions. These findings underscore anemias, IDA, are preventable and treatable contributors to pediatric stroke, although persistent nutritional deficiencies and limited supplement accessibility make addressing this a challenge for LMICs.

#### 4.2.3. Vasculopathies

Arteriopathies are among the most frequently reported causes of stroke in Asian and African regions. In a recent population-based cohort study in China, arteriopathy was identified in 49% of 700 cases of pediatric AIS, making it the most common risk factor.^40^ However, the true prevalence of arteriopathy in LMICs may be underestimated due to limited access to vascular imaging. In HICs, reported rates of vascular imaging in pediatric stroke cases varied by country, with Australia (91%), Canada (81%), Europe (90%), and the United States (83%). This is a stark contrast to reported vascular imaging in Asia (55%) and South America (52%).^17^ This diagnostic disparity likely contributes to underreporting of arteriopathy in resource-limited settings.

Moyamoya disease/syndrome is one of the most important arteriopathies globally and a particularly prominent cause of pediatric stroke in East Asia. “Moyamoya disease” typically refers to the idiopathic form, whereas “moyamoya syndrome” denotes cases associated with other conditions such as neurofibromatosis, SCD, Down syndrome, or prior cranial irradiation.^18^ MMD is the most common pediatric cerebrovascular disease in East Asian populations, with incidence rates of 0.94 per 100,000 in Japan and 2.3 per 100,000 in South Korea, the highest reported worldwide.^41,42^ It accounts for more than 6% of pediatric strokes globally.^18^

Across Asia, numerous studies identify MMD as the leading risk factor for AIS. Studies from Taiwan, India, the UAE, Thailand, and China consistently report moyamoya as the most common etiology.^3,8,24,39,43,44^ In Taiwan’s largest national pediatric stroke study, MMD accounted for 7.6% of ischemic strokes and was a major contributor to transient ischemic attacks.^2^ In China, the leading causes were arteriopathies including moyamoya (118 patients) and dissection (5 patients) for AIS, whereas and arteriovenous malformation (AVM) and cavernous malformation (CM) for were leading causes of hemorrhagic stroke.^44^ MMD was identified in 57.6% of children with arteriopathy in China and 11.5% in Iran. In Thailand, it constituted 11% of AIS cases, making it the most common etiology of ischemic stroke in these studies.^11^ Malaysian data similarly identify MMD as the leading risk factor.^45^ These etiologic patterns illustrate a marked contrast with Western countries, where arteriopathy profiles differ significantly.

MMD is also a known complication of SCD. Among Sudanese children with SCD presenting with stroke, 48 of 50 appropriately imaged patients had moyamoya, and 55 total children were diagnosed. This study shows a likely underestimate due to limited diagnostic resources.^38^ Thus, MMD represents both a regional genetic risk factor in East Asia and an important sequela of SCD.

Other vasculopathies in Asian and African regions include both infectious and noninfectious etiologies. Tuberculosis arteritis accounted for 47% of secondary vasculitis cases in the Philippines, while a study from Southern Khorasan (Iran) identified meningoencephalitis-induced vasculopathy as the most common cause of pediatric ischemic stroke.^6,11^ Mineralizing angiopathy, a noninfectious arteriopathy frequently associated with prior viral illness, was also reported.^24^ AVMs were the second most common vasculopathy overall, although particularly in hemorrhagic stroke.^2,39^

#### 4.2.4. Cardiac Disorders

A global analysis identified heart disease as the leading etiology of pediatric stroke in Asia and Africa (78.5%).^46^ Cardiac causes include rheumatic heart disease (RHD) and a broad spectrum of congenital heart diseases (CHD). In Asian children, congenital malformations with intracardiac shunting, cardiomyopathies, arrhythmias and endocarditis are predominant etiologies of ischemic stroke.^19^ Cardiac disease has similarly been reported as a key risk factor in Egyptian children.^47^ In Turkey, cardiac disorders represented the most common risk factor (17%) among 36 AIS cases and in Pakistan, 39% of pediatric stroke cases had an identifiable cardiovascular disorder, aligning with global findings and emphasizing the need for targeted cardiovascular assessment in pediatric stroke workups.^24,39,46^

In East Asia, patterns vary by country. Cardiac disease is an important cause of pediatric stroke in Hong Kong, whereas a 2007 retrospective study from Taiwan found cardiac etiologies to be less common, differing from Western cohorts in North America and Europe, where cardiac disorders account for 12-18% of pediatric AIS cases.^43^ In China, cardiac disease was reported the third most common cause of pediatric stroke (35%).^40^

RHD remains a particularly significant cardiac risk factor in LMICs. In Thailand, RHD accounted for 6% of childhood AIS cases.^39^ High rates of RHD, along with cardiomyopathy and myocarditis, illustrate the ongoing burden of infectious and post-infectious cardiac disease in developing countries.^20,46^

#### 4.2.5. Prothrombotic Disorders

Prothrombotic states, whether acquired or congenital, can also put children at greater risk for stroke. Prothrombotic states include deficiencies in protein C, protein S, and antithrombin III, as well as inherited thrombophilias, such as Factor V Leiden and MTHFR polymorphisms. One study reported prothrombotic disorders in 13% of pediatric stroke cases.^43^ In Egypt, a prospective study identified Factor V Leiden in 25% of children with ischemic stroke, and another Egyptian cohort found that 65% of affected children carried at least one inherited thrombophilic abnormality.^47^ Similarly, prothrombotic disorders accounted for 31.7% of AIS cases in Saudi children.^11^ In contrast, an Indian study reported a lower prevalence (<8%), attributing this difference to the substantial contribution of infectious etiologies to pediatric stroke in Asian populations compared with Western cohorts, where 30-76% of cases involve thrombophilia.^3^

Methylenetetrahydrofolate reductase (MTHFR) polymorphisms are particularly relevant in South and Southeast Asia. The MTHFR c.677C>T variant is the most prevalent mutation in Sri Lanka and other South Asian populations, and Egyptian studies likewise report an increased frequency of MTHFR polymorphisms among children with stroke.^11,47^ In Turkey, MTHFR variants were the third most common risk factor, present in 10% of pediatric stroke cases. In China, thrombophilia ranked as the second most common etiology (35%) among children with AIS.^40^

Autoimmune disorders also contribute to hypercoagulability. Systemic lupus erythematosus (SLE) is a well-recognized cause of pediatric stroke through inflammatory and prothrombotic mechanisms. In a South African cohort of children with SLE, 8.6% had evidence of cerebrovascular disease.^48,49^

#### 4.2.6. Other Considerations

Less commonly reported etiologies of pediatric stroke in Asian and African regions include genetic and metabolic disorders, trauma, and perinatal/neonatal factors. In one study, metabolic disorders accounted for 18% of risk factors, trauma for 11%, and mitochondrial disease for 6% of cases.^43^ Trauma-related mechanisms are variably represented across regions. Trauma accounted for 8% of risk factors in a Turkish cohort and was the most common etiology in a study from Thailand.^23,50^ Post-traumatic arterial dissection contributed to 10% of childhood AIS cases in India and 2.9% in Saudi Arabia.^11^ A Nairobi-based study identified connective tissue disorders as the most common major risk factor with a genetic basis, highlighting the need for expanded evaluation of hereditary arteriopathies in African cohorts.^4^ In Pakistan, 73.5% of children with childhood primary angiitis of the CNS (cPACNS) presented with ischemic stroke, and in Singapore, collagen vascular disease was identified in 33% of AIS cases. Elevated homocysteine levels were reported in 8 of 95 Indian children with AIS, emphasizing the contribution of metabolic disorders such as homocysteinemia.^11^

Perinatal and neonatal risk factors were described only briefly among Asian regions. Compared to older children, hemorrhagic strokes are more common in this population. One regional study identified perinatal asphyxia and neonatal sepsis as the most common causes of neonatal stroke, while another found dehydration and congenital heart disease to be more common among neonates compared with older children.^18,51^

Hemorrhagic stroke risk factors also vary by region. In a Turkish study of 22 children with hemorrhagic stroke, vitamin K deficiency accounted for 17 cases. The combination of low neonatal vitamin K levels, absence of postnatal supplementation, and reduced availability from breastfeeding explain the likely pathogenesis of vitamin K-dependent bleeding after 1 month of life.^52^ These findings underscore the broad spectrum of less frequent but clinically significant etiologies that contribute to pediatric stroke in low- and middle-income settings.

## 5. Clinical Features & Diagnostics

### 5.1. Clinical Symptoms in Asian Cohorts

Across studies, pediatric stroke most commonly presents with focal neurological deficits, particularly hemiparesis/hemiplegia, which is consistently the dominant clinical feature. Facial weakness and other focal signs (e.g., aphasia/dysphasia) frequently accompany these deficits. Seizures are the second most common presentation, occurring in approximately 20–58% of patients, and are especially prominent in younger children and neonates, where they may be the initial or sole presenting symptom.^24,44^ Altered mental status (including encephalopathy or decreased consciousness) is also common, particularly in more severe cases, and may overlap with diffuse neurological signs.

In addition to focal deficits, nonspecific systemic and neurological symptoms are frequently reported such as headache, vomiting, fever, lethargy or poor feeding, especially in neonates. Less common but notable features include hydrocephalus, hypertension, neck stiffness, and cardiorespiratory dysfunction. Predominant symptoms in neonates and infants were seizures, lethargy, poor feeding and more nonspecific signs, most likely attributed to age. Older children and adolescents more commonly presented with hemiparesis/hemiplegia with focal deficits.

### 5.2. Clinical Symptoms in African Cohorts

Across African studies, pediatric stroke most consistently presents with motor deficits, particularly hemiparesis/hemiplegia, similar to Asian studies, which is nearly universal in some cohorts (up to 95%) and often the dominant presenting feature, especially in children aged 5-10 years. Speech and cranial nerve deficits are also prominent with aphasia and facial nerve involvement. Seizures are a common but less consistent feature mentioned among articles, while altered mental status is observed in both younger and older children, often indicating more severe or diffuse involvement. Additionally, headache and visual disturbances have been reported. Younger children, less than 5 years of age, are more likely to present with seizures, altered mental status, or mixed/nonspecific symptoms, while older children commonly present with hemiparesis and focal deficits, also similar to Asian cohorts.

### 5.3. Clinical Presentation Impact

Pediatric stroke presents with a wide spectrum of clinical symptoms that vary by age and region. Across Asian and African cohorts, presentations range from focal neurological deficits to nonspecific systemic signs, often complicating timely recognition. While common symptoms have been identified, including seizures, focal neurologic deficits (particularly hemiparesis, as well as speech and visual disturbances), headache, and impaired consciousness, additional features such as ataxia, altered mental status, and language disturbances are also frequently reported. The nonspecific and heterogeneous nature of these presentations, along with the presence of stroke mimics such as reversible posterior encephalopathy syndrome, intracranial infection, inflammatory disease, and tumors, contributes to delays in diagnosis and treatment. This diagnostic challenge is especially significant given the substantial morbidity associated with pediatric stroke, as up to 80% of affected children develop long-term motor impairments or cognitive deficits, and approximately 15% subsequently develop epilepsy.^10,53^ Understanding these clinical patterns is essential to improve early recognition and management, particularly in both high- and low-resource settings.

### 5.4. Diagnostic Imaging

Many established protocols, primarily among HICs, recommend MRI as the diagnostic gold standard due to its superior sensitivity for early ischemia and ability to evaluate vascular etiologies. However, Asian and African regions are challenged with limited access to MRI and even CT. The IMAGINE database reports an average of 2.61 CT scanners and 1.06 MRIs per million people (pmp) in Africa, with several countries noted as having no CT or MRI.^54^ Asian countries fared better at 9.97 CT scanners and 4.31 MRIs pmp but were still much lower than the European average of 22.07 and 19.73 pmp, respectively.^54^ This limited access is further complicated by differences between countries as a study on imaging capacity in Tanzania noted that their public health sector had less than 5% of the CT and less than 20% of the capacity of South Africa’s healthcare system.^55^ Distribution continues to be unequal within countries, with studies in Ghana and Tanzania reporting the majority of their advanced imaging technology being available near their largest cities.^55,56^ A needs assessment of MRI access in Africa also notes that the majority of MRIs were low-field strength or even obsolete.^57^ Given the necessity of advanced imaging equipment for timely diagnosis, disparity in available technology can delay care beyond six hours, leading to greater rates of complication and comorbidities for patients. Access to MRI for appropriate angiography studies is also crucial for determining the presence of arteriopathies that could place a patient at risk for recurrent ischemic events.^58^

## 6. Management Strategies

### 6.1. Current Treatment Recommendations

Treatment guidelines for pediatric stroke remain challenging and often controversial due to the scarcity of clinical trials and limited evidence regarding treatment safety, efficacy, and long-term outcomes in children. Current guidelines vary by institution, region, and nation. As a result, current management practices vary widely across institutions, regions, and countries, as summarized by Table 3. In 2019, the American Heart Association/American Stroke Association (AHA/ASA) released updated recommendations addressing key aspects of pediatric stroke care, including eligibility for thrombolytic therapy, considerations for mechanical thrombectomy, application of the Pediatric NIH Stroke Scale, supportive management, and etiology-specific treatment pathways.^59^ In 2021, a collaborative analysis by the International Pediatric Stroke Study (IPSS) and the Pediatric Neurocritical Care Research Group evaluated 47 surveys and found that at least 41 pediatric centers in the United States and Canada utilized formal acute stroke protocols. Their recommendations emphasized rapid neuroimaging, preference for MRI-based imaging over CT when feasible, and the importance of a multidisciplinary stroke response team.^60^

**Table 3.** Summary of Pediatric Stroke Management Strategies, Guideline Recommendations, and Regional Practice Variability Across Asia and Africa

Category	Stroke subtype/Etiology	Recommended management	Key guideline/Evidence	Regions mentioned	LMIC-relevant considerations
General Acute Management	Acute Ischemic Stroke (AIS)	Stabilization, neuroprotective care, management of BP, hyperglycemia, fever, cerebral edema, and seizures	AHA/ASA 2019 recommendations	Non-specific	Limited ICU access, delayed presentation, and inconsistent stroke pathways
Intravenous Thrombolysis (IVT)	AIS	Alteplase within 4.5 hours in selected patients aged 28 days–18 years	2026 AHA/ASA pediatric AIS guideline	Non-specific	Requires rapid imaging, stroke expertise, and protocolized systems of care
Endovascular Thrombectomy (EVT)	AIS	EVT may be reasonable in carefully selected children aged ≥6 years	2026 AHA/ASA guideline; Save ChildS registries	Non-specific	Limited by lack of neurointerventional centers and transfer networks
Antithrombotic Therapy	Non-SCD AIS	Aspirin (1–5 mg/kg/day) or anticoagulation followed by long-term aspirin therapy	American College of Chest Physicians recommendations	Non-specific	Medication access and monitoring capacity vary substantially
Hemorrhagic Stroke Management	Hemorrhagic Stroke	Coagulopathy correction, seizure prophylaxis, ICP management, surgical intervention when indicated	2019 AHA/ASA Scientific Statement	Non-specific	Neurosurgical expertise often concentrated in tertiary urban centers
Acute-phase AIS Management	AIS	Variable use of LMWH, aspirin, antiepileptics, supportive care, and selective thrombolysis	Regional cohort studies	Turkey, UAE, China, Malaysia, Cameroon	Significant variability reflects differences in imaging access, institutional protocols, and provider expertise
Surgical Management	Hemorrhagic stroke/structural lesions	Surgical intervention for hydrocephalus, meningeal hemorrhage, brain tumors	Regional cohort studies	Guinea (Conakry)	Severe neurosurgical limitations and need for medical evacuation reported
Infectious Etiology Management	Infection-associated AIS	Pathogen-specific antimicrobial therapy; IVIG explored in selected cohorts	China cohort; WHO-aligned infectious management	China, India	High infectious burden influences stroke prevention and treatment priorities
Sickle Cell Disease (SCD)	SCD-related stroke	Urgent transfusion, chronic transfusion therapy, hydroxyurea (HU)	Randomized trial evidence; international SCD guidelines	Uganda, Sudan, Sub-Saharan Africa	HU increasingly important where chronic transfusion is impractical
Moyamoya Disease (MMD)	MMD-related stroke	Surgical revascularization; anticoagulation generally avoided	AHA/ASA recommendations; Korean meta-analysis	South Korea, East Asia	Limited pediatric neurosurgical expertise outside tertiary centers
Cardiac/Prothrombotic/Genetic Conditions	Etiology-specific stroke	Early screening, monitoring, caregiver/provider education	Expert consensus recommendations	Non-specific	Early diagnosis frequently limited by resource availability and specialist access

Abbreviations: AIS = arterial ischemic stroke; AHA/ASA = American Heart Association/American Stroke Association; BP = blood pressure; EVT = endovascular thrombectomy; HU = hydroxyurea; ICP = intracranial pressure; IVIG = intravenous immunoglobulin; IVT = intravenous thrombolysis; LMIC = low- and middle-income country; LMWH = low-molecular-weight heparin; MMD = moyamoya disease; SCD = sickle cell disease; UAE = United Arab Emirates.

Most recently, the updated 2026 AHA/ASA stroke guideline made several recommendations for pediatric stroke care for the first time, although limited to AIS.^58^ Strategies include intravenous thrombolysis (IVT), Endovascular Thrombectomy (EVT), and antithrombotic therapy, specifying based on age. IVT with alteplase is recommended in pediatric patients aged 28 days to 18 years old with confirmed AIS presenting within 4.5 hours; EVT, informed by the Save ChildS and Save ChildS Pro registries, may be reasonable in carefully selected children aged ≥6 years, with more limited evidence and greater technical complexity in younger children; systematic evidence remains lacking for neonates. [source]. The rationale for the age 6 cutoff is based on anatomic studies showing that intracranial vessels in children ≥6 years are nearly adult-sized, whereas younger children have smaller caliber access and cerebral vessels posing greater procedural challenges.

Additional guidelines address specific stroke subtypes. The primary AHA guidance on pediatric hemorrhagic stroke remains the 2019 AHA/ASA Scientific Statement on Management of Stroke in Neonates and Children.^59^ Recommendations include coagulopathy correction, seizure prophylaxis, ICP management, and surgical intervention when applicable. For non–SCD-related pediatric arterial ischemic stroke, the American College of Chest Physicians recommends initiating anticoagulation or aspirin therapy (1-5 mg/kg/day) until arterial dissection or cardioembolic causes are excluded, followed by long-term aspirin therapy for at least two years.^55^ Despite these efforts, substantial gaps remain in translating recommendations into clinical practice, and significant variability persists across healthcare systems worldwide. These global challenges highlight the need to contextualize pediatric stroke management within LMICs, particularly in Asia and Africa, where resource constraints, delayed diagnosis, and limited access to neuroimaging and subspecialty care significantly influence treatment feasibility and outcomes.

### 6.2. Acute-Phase Management

Immediate stabilization and supportive management remain the cornerstone of AIS care in children. Recommended strategies include the management of blood pressure, hyperglycemia, fever, cerebral edema, and seizures. Acute treatment of childhood AIS focuses on neuroprotective management by maintaining cerebral perfusion and reducing metabolic demand from fevers and seizures.^61^ The use of thrombolytics in children remains controversial; most recommendations are extrapolated from adult studies, even though the pathophysiology of thrombus formation has been theorized to differ between children and adults and should be considered on a case-by-case basis.

Reported practice patterns across Asia and Africa demonstrate considerable variability:

Turkey: Acute management was primarily symptomatic. Low-molecular-weight heparin (LMWH) (1.5 mg/kg q12h for infants ≤2 months; 1 mg/kg q12h for ages 2 months-18 years) was used in 11 patients, and aspirin (2-3 mg/kg/day) was used as prophylaxis in 33 patients.^52^

United Arab Emirates: Among 17 medically managed patients, 8 received aspirin alone and 4 received aspirin plus standard-dose LMWH after excluding hemorrhagic stroke. LMWH was continued for 3-6 months, while neonates received only anti-seizure therapy and supportive care.^8^

China: Management approaches varied across studies. One cohort reported 56 children (37.5%) receiving intravenous thrombolytic therapy after confirming normal coagulation parameters, with clinical improvement in all but one child.^23^ Other studies described exclusively conservative management, including intracranial pressure–reducing agents, vasodilators, nutritional neuromodulatory therapy, anti-inflammatory agents, and antivirals, with very limited use of antiplatelets or anticoagulants. In one series, only 5.23% of AIS patients were discharged on aspirin, 1.16% on clopidogrel, and 1.16% on warfarin.^44^ Another study reported that 49% received antiepileptic therapy, 40% aspirin, and 26% LMWH.^24^

Malaysia: Half of the pediatric stroke population (38 patients, 50%) received medical therapy with either antiplatelet or anticoagulant agents.^45^

Cameroon: Thrombolysis may be considered within a 4.5-hour window on a case-by-case basis when an arterial infarction is promptly diagnosed.^62^

Guinea (Conakry): Of 38 children, 76.3% received medical management, while 23.7% underwent surgical intervention. Surgical indications included post-traumatic meningeal hemorrhage, hydrocephalus, and brain tumors. However, several children required medical evacuation abroad and subsequently died, highlighting severe resource limitations.^63^

These examples suggest that reported management strategies often reflect available infrastructure rather than standardized regional practice. Acute-phase pediatric stroke management in LMICs is shaped by resource availability, diagnostic capacity, provider expertise, and institutional protocols. Practice patterns vary widely, not only between countries but also across hospitals within the same region, similar to the variability observed among HICs. This underscores the urgent need for standardized, resource-adapted pediatric stroke protocols in Asia and Africa.

### 6.3. Etiology-Specific Management

When an underlying stroke etiology is identified, treatment should be directed at the primary cause. In the context of infectious etiologies, pathogen-specific therapy is essential. One study in China administered intravenous immunoglobulin (IVIG) to children presenting with fever, drowsiness, and neurological dysfunction related to infection. All treated patients survived, an important finding given the typically high mortality in this population. The authors hypothesized that IVIG exerted vasodilatory and anti-inflammatory effects, reducing local vascular pathology and improving neurological outcomes in AIS.^64^ Other CNS infections, such as meningitis, should be managed according to established national and WHO-aligned protocols using locally available antimicrobial therapies.

Given the high prevalence of malaria, sepsis, and other infectious precipitants, a study from India suggested that hydroxyurea (HU) may provide added benefit in certain regions by offering anti-infectious prophylaxis, thereby lowering the risk of infection-triggered strokes.^65^ However, in many LMICs, antiplatelet agents such as aspirin have not demonstrated consistent benefit in primary stroke prevention among children with infection-related vasculopathies.^20^ Strengthening vaccine uptake and public health education remains critical in regions where infections are endemic.

#### 6.3.1. Sickle Cell Disease

SCD is the only pediatric stroke risk factor with randomized controlled trial evidence-based management. Current guidelines recommend urgent blood transfusion for any child with SCD and acute neurological symptoms, with the goal of reducing HbS to <30% and raising total hemoglobin to 10-11 g/dL. For secondary stroke prevention, regular chronic transfusions are advised to maintain pre-transfusion HbS levels <30% and pre-transfusion hemoglobin >9 g/dL, via simple or exchange transfusions.^20^

HU is increasingly recognized as a practical and effective therapy in LMICs. A Ugandan trial advocated for early and continuous HU therapy to prevent cerebrovascular injury, noting its feasibility and efficacy in reducing SCD morbidity.^33^ In Sudan, most patients received HU according to guideline-based dosing (starting at 10 mg/kg/day, titrated to a maximum of 35 mg/kg/day, with most children receiving 15 mg/kg/day). In this study, more than two-thirds of patients received blood transfusions, with 13.9% undergoing chronic blood transfusions every month for 3 years to prevent primary or secondary stroke.^66^

#### 6.3.2. Moyamoya Disease

In MMD, anticoagulation is generally not recommended because of the heightened risk of intracerebral hemorrhage. Surgical revascularization is the primary treatment.^64^ Evidence for medical therapy remains limited, as no randomized trials guide pharmacologic decision-making. A Korean meta-analysis of 9 studies enrolling 16, 186 patients with moyamoya (adult and children) found that antiplatelet therapy reduced the risk of hemorrhagic stroke, but did not reduce ischemic stroke or improve functional outcomes. Antiplatelet agents evaluated included aspirin, clopidogrel, and cilostazol; while cilostazol shows promise in adult MMD, evidence is limited for use in children.^67^

#### 6.3.3. Children With Cardiac, Hematologic, Prothrombotic, Genetic, or Metabolic Conditions

For children with known congenital heart disease, hematologic disorders, prothrombotic conditions, or genetic/metabolic abnormalities, early screening at diagnosis is essential. Ongoing clinical monitoring and prompt evaluation of new neurological symptoms can facilitate early detection of stroke. Provider and caregiver education plays a critical role in ensuring timely recognition and intervention in these high-risk populations.

## 7. Discussion

### 7.1. Epidemiologic Disparities

The prevalence and incidence of pediatric stroke are highest in Asia and Africa, reflecting both region-specific disease burdens and structural inequities. Although absolute inequalities in pediatric stroke burden have narrowed over time, relative inequalities continue to widen, largely due to slower progress in low-SDI regions. These widening gaps underscore the need for targeted, equitable resource allocation to communities most disproportionately affected.^68^ Children in LMICs also experience higher mortality rates, driven by late presentation, limited access to imaging and expertise, and broader health-system constraints.

Regional differences in disease prevalence further shape the epidemiologic landscape. Several Asian and African subregions have higher rates of stroke-causing conditions like SCD, MMD, TBM, and congenital infections. This indicates the need for heightened vigilance and focused research in these areas. Genetic variability within and between countries also presents opportunities for future studies, including investigations into genetic predisposition and potential protective variants in regions with lower-than-expected stroke incidence. Given the geographic diversity and data gaps across Asia and Africa, the establishment of institutional, national, and international pediatric stroke registries represents a critical avenue for improving epidemiologic surveillance and closing existing knowledge gaps.

### 7.2. Etiologic Differences

Across Asia and Africa, the most common etiologies of pediatric stroke include infectious diseases, SCD, and MMD. These patterns differ from HICs, demonstrating the importance of region-specific stroke surveillance and of maintaining a high index of suspicion when children with known risk factors present with acute neurological deficits. Strategies such as vaccination, early diagnosis, and timely treatment of endemic infections can substantially reduce childhood stroke burden in these regions.^69^

### 7.3. Diagnostic & System-Level Disparities

Diagnosing pediatric stroke remains a global challenge in both low- and high-income settings due to its heterogeneous clinical presentation, which varies by age, stroke subtype, and disease severity. Symptoms are often nonspecific and may mimic more common pediatric conditions, contributing to delayed recognition and underscoring the need for heightened clinical suspicion across healthcare settings.

Diagnostic disparities are particularly evident in neuroimaging access. In many LMIC settings, CT remains the primary imaging modality because of lower cost, greater availability, faster acquisition times, and reduced sedation requirements in children. In contrast, MRI, which is more commonly utilized in HIC pediatric stroke protocols because of its superior sensitivity for early ischemia and vascular etiologies, is often limited by high installation and maintenance costs, shortages of specialized personnel, longer scan times, and restricted availability outside tertiary urban centers. Consequently, reliance on CT remains common across many Asian and African regions, potentially contributing to delayed diagnosis, underrecognition of arteriopathies, and incomplete etiologic evaluation.

Beyond imaging limitations, broader systems-level inequities further widen diagnostic gaps. Findings from the Global Alliance for Pediatric Stroke Epidemiology and Resources survey demonstrated substantial shortages of pediatric stroke expertise and formal training programs across many Asian and African regions. Institutional pediatric stroke protocols were also far less common in LMIC settings compared with North American and European centers. Furthermore, many HIC-derived pediatric stroke pathways rely on advanced imaging, subspecialty availability, and neurointerventional resources that may not be consistently accessible in resource-limited environments. Together, these disparities likely contribute to delayed diagnosis, increased neurologic morbidity, recurrent stroke risk, and poorer long-term outcomes among children in LMICs. Addressing these inequities will require expansion of imaging infrastructure, development of resource-adapted pediatric stroke pathways, increased workforce training, and implementation of scalable diagnostic algorithms tailored to varying healthcare capacities.

### 7.4. Management Variability

Asian and African regions share many of the global challenges in pediatric stroke management, particularly the limited availability of pediatric clinical trial data to guide evidence-based use of reperfusion therapies such as intravenous thrombolysis and mechanical thrombectomy. However, treatment disparities are often more pronounced in LMIC settings, where resource limitations substantially affect the feasibility of implementing current guideline-based recommendations. This is particularly evident in sickle cell disease (SCD)-related stroke management. Although chronic transfusion therapy is recommended for secondary stroke prevention in children with SCD, limited access to safe blood products, inconsistent transfusion infrastructure, and concerns regarding iron overload restrict implementation in many resource-limited regions. As a result, hydroxyurea has emerged as an important alternative strategy in settings where chronic transfusion therapy is unavailable, unsafe, or impractical.^11^

Availability of hyperacute stroke interventions also varies considerably across regions. Intravenous thrombolysis was reportedly offered to eligible pediatric patients by 42.4% of respondents in Europe and North America compared with only 3.7% in South America and 19.0% in Asia and Africa.^70^ Similarly, hyperacute recanalization therapies, including endovascular mechanical thrombectomy and chemical thrombolysis with agents such as alteplase or tenecteplase, were most commonly available in Europe and North America. Standardized pediatric stroke pathways also remain inconsistently implemented worldwide. Institutional pediatric stroke protocols were reported in 55% of Europe/North America centers, compared with 44.5% of South American centers and only 24% of centers in Asia and Africa, while formal national or regional pediatric stroke guidelines were even less common.^70^

Recent AHA/ASA pediatric stroke recommendations further emphasize that hyperacute interventions such as IVT and EVT should ideally be performed in centers with pediatric stroke expertise and access to interventionalists experienced in pediatric endovascular procedures. However, many healthcare systems across Asia and Africa lack pediatric stroke referral networks, neurointerventional infrastructure, organized stroke response pathways, and telestroke capabilities necessary to support these time-sensitive therapies. Consequently, children in many LMIC settings may face delayed diagnosis, delayed transfer to specialized centers, or complete inaccessibility of advanced reperfusion therapies.

Although expert consensus statements from organizations such as the American College of Chest Physicians and the AHA/ASA provide an important framework for pediatric stroke management, translation of these recommendations into clinical practice remains inconsistent even in high-resource settings. Within LMICs, access to reperfusion therapy and protocolized care is often concentrated in tertiary urban centers, while non-tertiary facilities may lack imaging, pediatric neurology consultation, neurocritical care, or transfer pathways needed to deliver time-sensitive treatment. Furthermore, most current treatment algorithms are derived from HIC healthcare systems and often lack tiered, resource-adapted approaches that account for limitations in neuroimaging, subspecialty availability, blood product access, and healthcare workforce capacity. Future pediatric stroke guidelines should incorporate scalable, resource-sensitive management pathways tailored to varying healthcare infrastructures to improve feasibility, implementation, and equity in stroke care globally.

### 7.5. Geographic and System-Level Axes of Pediatric Stroke Care

Across studies, pediatric stroke care differs substantially between LMICs and HICs across epidemiology, diagnostic capacity, treatment availability, and clinical outcomes. LMICs, particularly across Asia and Africa, carry a disproportionately higher pediatric stroke burden driven by infectious diseases, sickle cell disease, moyamoya disease, rheumatic and congenital heart disease, and delayed recognition of vascular risk factors. In contrast, HICs generally report lower incidence rates and different etiologic distributions, with greater identification of arteriopathies, cardiac disorders, and genetic or inflammatory causes due in part to broader access to advanced diagnostic evaluation.

Diagnostic infrastructure represents one of the clearest divides between healthcare settings. HIC pediatric stroke systems commonly utilize MRI-based, protocolized, multidisciplinary care pathways supported by pediatric neurologists, neuroradiologists, neurocritical care teams, and stroke response protocols. Conversely, many LMIC settings rely primarily on CT-based imaging or have limited imaging access altogether, contributing to delayed diagnosis, underrecognition of etiologies, and reduced eligibility for time-sensitive interventions. Treatment availability similarly varies, with HIC centers more frequently implementing reperfusion therapies, endovascular thrombectomy, formal stroke pathways, and specialized neurocritical care, whereas many LMIC regions depend on supportive management and resource-adapted treatment strategies. These disparities contribute to increased morbidity, mortality, delayed rehabilitation access, and poorer long-term neurologic outcomes in resource-limited settings.

Long-term care represents another major axis of disparity. Pediatric stroke survivors often require rehabilitation, neuropsychological support, epilepsy management, educational accommodations, and caregiver support. In LMIC settings, access to these services may be limited outside tertiary centers, contributing to persistent disability even after survival from the acute event.

Importantly, these inequities are not solely defined by differences between countries or income classifications. Pediatric stroke disparities in Asia and Africa can be conceptualized across two intersecting axes: geographic and systemic. At the geographic level, LMICs experience broader structural limitations in healthcare financing, imaging infrastructure, subspecialty workforce availability, and access to standardized pediatric stroke care compared with HICs. However, substantial heterogeneity also exists within LMIC healthcare systems themselves. Tertiary referral centers located in major urban areas may offer MRI, angiography, pediatric intensive care, stroke protocols, and multidisciplinary expertise comparable to HIC institutions. In contrast, secondary hospitals and rural healthcare facilities frequently face major limitations in imaging availability, pediatric neurology expertise, neurointerventional access, laboratory capacity, and timely referral pathways. As a result, children evaluated at non-tertiary centers may experience substantial delays in recognition, diagnosis, transfer, and treatment initiation before reaching specialized facilities, particularly in regions with limited emergency transport systems or fragmented referral networks.

This intra-country variability highlights that pediatric stroke outcomes are influenced not only by national income status but also by local healthcare infrastructure, regional resource distribution, and institutional capacity. Consequently, pediatric stroke care in many LMIC settings exists along a continuum rather than a binary distinction between “LMIC” and “HIC” care models. Recognizing these intersecting geographic and systemic disparities is critical for developing context-specific, resource-stratified pediatric stroke frameworks that incorporate scalable diagnostic pathways, referral networks, telestroke systems, workforce training, and tiered treatment algorithms adapted to varying levels of healthcare infrastructure. This framework suggests that pediatric stroke inequities should not be conceptualized solely through national income classifications, but through the interaction between regional disease burden, institutional capacity, and healthcare accessibility.

### 7.6. Current Efforts

While limitations exist globally, several emerging initiatives show promise for improving pediatric stroke care in LMICs. In Asia and Africa, areas for improvement include development of regional and national policies aimed at creating more facilities that can provide cost-effective standard of care and training programs for pediatric stroke (global alliance survey).Current efforts broadly fall into three categories: screening and community detection, workforce education and training, and prevention and research infrastructure. Specifically, in Ghana they aim to address screening challenges, clinicians adopted the Questionnaire for Verifying Stroke-Free Status (QVSFS), an inexpensive, simple, and accurate tool for identifying possible stroke cases in the community.^69^ Educational initiatives focused on SCD have also been introduced. For example, Ghafuri et al developed a training curriculum for healthcare professionals in low-income settings to improve SCD-related stroke prevention and care.^71^ Large-scale programs, such as the Stroke Prevention in Nigeria (SPIN) and (SPRING) trials, have advanced research capacity building, quality improvement, and government partnerships, establishing a sustainable model for primary stroke prevention in children with SCD in low-resource settings.^72^ Additionally, Tan et al proposed an algorithm tailored to diagnosing childhood AIS in resource-limited environments, providing a practical framework for regions lacking advanced imaging or subspecialty care.^11^ These efforts illustrate meaningful progress and highlight the direction for future work aimed at closing gaps in pediatric stroke care across Asia and Africa.

### 7.7. Strength and Limitations

This review provides a broad synthesis of pediatric stroke literature across Asia and Africa using a structured search strategy and the Global Burden of Disease regional framework, allowing for comparison of epidemiology, etiologies, clinical presentation, and management across diverse settings. By incorporating studies from multiple countries and highlighting region-specific risk factors such as infectious diseases, sickle cell disease, and moyamoya disease, this review provides a broad and clinically relevant perspective on pediatric stroke with emphasis on LMICs with high-burden regions. However, several limitations should be considered. Because much of the available literature comes from tertiary referral centers, findings may overestimate diagnostic and treatment capacity and underestimate delays and outcomes in rural or non-tertiary settings. As a narrative review without formal systematic methodology or risk of bias assessment, findings are descriptive and subject to selection bias. Additionally, restriction to English-language publications, indexed databases, and inclusion of grey literature introduces potential publication bias. Finally, the included studies are highly heterogeneous in design, definitions, and reported outcomes, limiting direct comparability. Data from Asia and Africa remain fragmented, with many studies derived from single-center or tertiary institutions, leading to underrepresentation of rural and lower-resource settings and potential underestimation of true disease burden.

## 8. Conclusion

This review highlights the substantial burden of pediatric stroke in Asia and Africa, the two regions with the highest global prevalence and incidence. Although clinical presentations are similar to those in high-income settings, delays in diagnosis and limited access to neuroimaging, specialized care, and standardized protocols contribute to poorer outcomes and highly variable management approaches.

To improve outcomes, targeted and actionable strategies are needed. Future progress will require not only expanding pediatric stroke resources globally but also building tiered systems of care that connect community, secondary, and tertiary centers through referral networks, training, and resource-adapted protocols. These include the development and implementation of resource-stratified diagnostic and treatment algorithms, expansion of regional and national pediatric stroke registries to improve epidemiologic surveillance, and investment in workforce training and multidisciplinary stroke teams. Strengthening access to essential diagnostics, particularly MRI and vascular imaging, alongside scalable tools such as clinical screening protocols, is critical for early recognition. Preventive strategies should prioritize infection control (e.g., vaccination, early treatment of CNS infections) and screening and management of regionally targeted high-risk conditions (e.g., SCD).

Future research should focus on multicenter, region-specific studies to better define epidemiology and outcomes, as well as clinical trials evaluating the safety, efficacy, and feasibility of acute and preventive therapies in pediatric populations within LMIC settings. Additionally, there is a need to develop and validate cost-effective, resource-adapted diagnostic tools and treatment protocols, as well as to explore genetic and environmental contributors to regional stroke risk. Addressing these priorities through coordinated global and local efforts will be essential to reducing disparities and improving outcomes for children with stroke in Asia and Africa.
